# Transvesical laparoendosopic single-site surgery as a valuable option to remove eroded materials from the bladder: single-center experience and a review of the literature

**DOI:** 10.1007/s11255-018-2039-y

**Published:** 2018-11-26

**Authors:** Maciej Przudzik, Michał Borowik, Mirosław Łesiów, Roman Łesiów

**Affiliations:** Department of Urology, Faculty of Medicine, University of Warmia and Mazury, Ul. Oczapowskiego 2, 10-719 Olsztyn, Poland

**Keywords:** Urinary incontinence, Surgery, Complications, Laparoendoscopic single-site surgery

## Abstract

**Introduction:**

Currently, polypropylene materials are used widely for the treatment of various urogynecologic disorders. This type of treatment can be complicated, although rarely, with erosions of the polypropylene implants into the bladder or the urethra. There is no established treatment for such complications. We present our experience in transvesical laparoendoscopic single-site surgery (T-LESS) removal of eroded materials, and a review of the literature in this field.

**Materials and methods:**

From June 2015 to May 2017 eight females, with an average age of 66.5 years (range 55–80 years), were referred to our Center because of the erosion of polypropylene material in the bladder, after anti-incontinence or pelvic organ prolapse treatment. Patients were diagnosed with ultrasound and cystoscopy. Seven bladder erosions and one bladder and urethral penetration were found. Patients were qualified for removal with the T-LESS approach. The Tri-Port + disposable set and standard laparoscopic instruments were used. The eroded materials were dissected and cut away, and the defects of the bladder wall were closed with barbed sutures. The peri-operative efficacy and safety of the method were assessed, and the patients were scheduled for follow-up visits at 6 weeks and every 3 months thereafter. The patients were offered a cystoscopic exam during the 7–10 month period after the operation.

**Results:**

The procedures were completed successfully in all patients. No blood loss or complications were observed. The mean operative time was 54.5 min, and the average hospital stay was 30 h. During a follow-up at 11 months, all patients were cured, except for one who presented urethral erosion.

**Conclusions:**

The T-LESS technique for removal of eroded meshes is a safe and effective method. The precise access to the bladder minimizes morbidity, and suturing the bladder wall defects may reduce the risk of recurrence.

## Introduction

The use of polypropylene materials has been a standard treatment for stress urinary incontinence and pelvic organ prolapse since 1995, when Ulmsten and Petros introduced a fabric TVT tape to perform mid-urethral placement of a sling. This revolutionized urinary incontinence therapy [1]. The high success rate of mid-urethral slings encouraged surgeons to repair pelvic organ prolapse with the use of a polypropylene mesh [2].

Although the long-term results are satisfactory, these procedures are associated with various complications even as serious as intestinal perforation [3]. The other types of complications include bladder perforation, vascular injury, urinary tract infection, urinary retention, de novo detrusor over-activity, chronic pelvic pain, and mesh erosion into the vagina, urethra, or bladder [4–8].

The presence of the eroded materials in the bladder demands careful consideration regarding the best treatment option. The overall reported incidence of tape and mesh erosion into the bladder is low (around 0.5–0.6%) [9]. The biggest challenge among the complications is bladder erosions resulting from not following the gold standard for repair surgery, and thus the relapse of the disease. Repair operations can be performed by open or endoscopic transurethral or laparoscopic techniques. Currently, open approaches are the most commonly used methods.

Disadvantages of standard methods, either open, laparoscopic, or robotic, include relatively high morbidity, a long hospital stay and unsatisfactory cost-effectiveness [9, 10]. Therefore, a logical implication was to use other minimally invasive procedures, either transperitoneal or transvesical single-port techniques that were introduced in urology by Rane et al. and Ingber et al. in 2008–2009 [11, 12]. Here we present our clinical results in eight patients undergoing surgery using the transvesical, laparoendoscopic, single-site surgery (T-LESS) to remove surgical materials that had eroded into the bladder. The literature on the application of one-port surgery for the treatment of erosions is scarce. Therefore, we reviewed and compared the results of our group with material presented by other investigators.

## Materials and methods

**Fig. 1:**
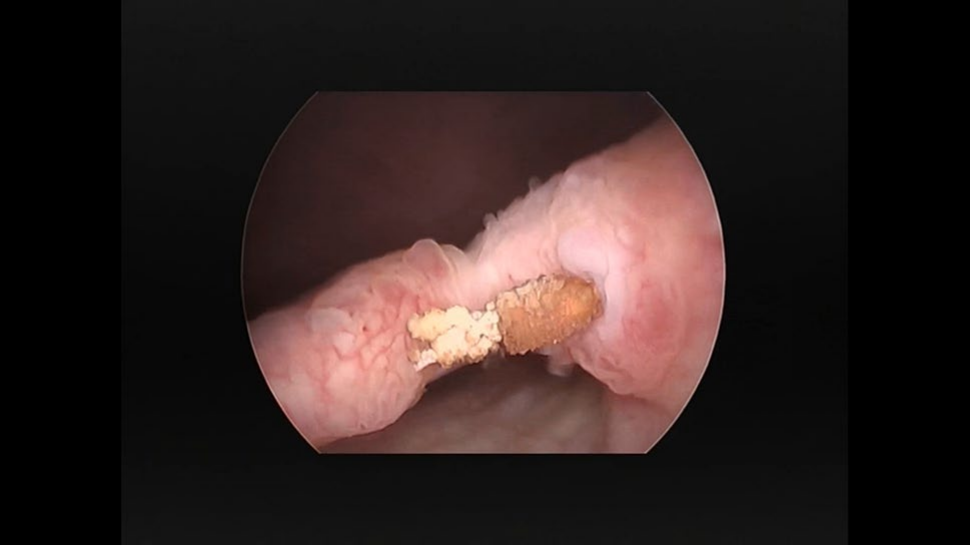
Exemplary eroded tape removed from patients

From June 2015 to May 2017, eight women, aged 66.5 years (range 55–80), with an average BMI of 28.4 (21.09–42.75) were referred to our Center due to bladder erosion of the polypropylene material after anti-incontinence or pelvic organ prolapse treatment (Fig. 1). One woman presented with persistent, stress urinary incontinence (SUI), and in two patients the symptoms of de novo urgency were diagnosed.

Two patients underwent unsuccessful transurethral treatment. The detailed patient data are presented in Table 1. Regardless of the material, and its size and location in the bladder, all patients were qualified for surgery with a single-port technique. We chose this technique because of the Center’s experience and a significant reduction in the time of surgery relative to the standard laparoscopic technique.

**Table 1:** Demographic data and outcomes

	Age	BMI	Initial surgery	Symptoms (type)	Urine culture	Category time-site classification	Access	Operation time (min)	Compli-cations	Post-op stay (h)	Post-op bladder catheterization (days)	Post-op VAS 1 day	Follow-up (months)
Pt1	62	26.40	TVT (the name unknown)	Urgency	Neg	4b/T3/S3	Tri-Port+	135	None	96	9	6	32
Pt2	79	25.71	Calistar	Urgency Pelvic pain	Neg	4b/T3/S3	Tri-Port+	50	None	19	6	2	21 UTI (6)
Pt3	78	24.70	TVT (the name unknown)	Urgency Pelvic pain	Neg	4b/T3/S3	Tri-Port+	23	None	8	6	2	17
Pt4	59	29.77	TVT (the name unknown)	Mild urgency	Neg	4b/T3/S3	Tri-Port+	60	None	32	9	3	17
Pt5	61	33.46	TOT (the name unknown)	Mild urgency Pelvic pain	Neg	4b/T3/S3	Tri-Port+	42	None	23	5	2	13
Pt6	58	23.50	Calistar	Urgency UTI	*E. coli*	4b/T3/S3	Tri-Port+	75	None	27	6	3	11
Pt7	55	42.75	TVT (the name unknown)	Urgency Pelvic pain	Neg	4b/T3/S3	Tri-Port+	23	None	15	7	1	11
Pt8	80	21.09	TVT (the name unknown)	Mild urgency	Neg	4b/T3/S3	Tri-Port+	28	None	23	8	1	10

**Fig. 2:**
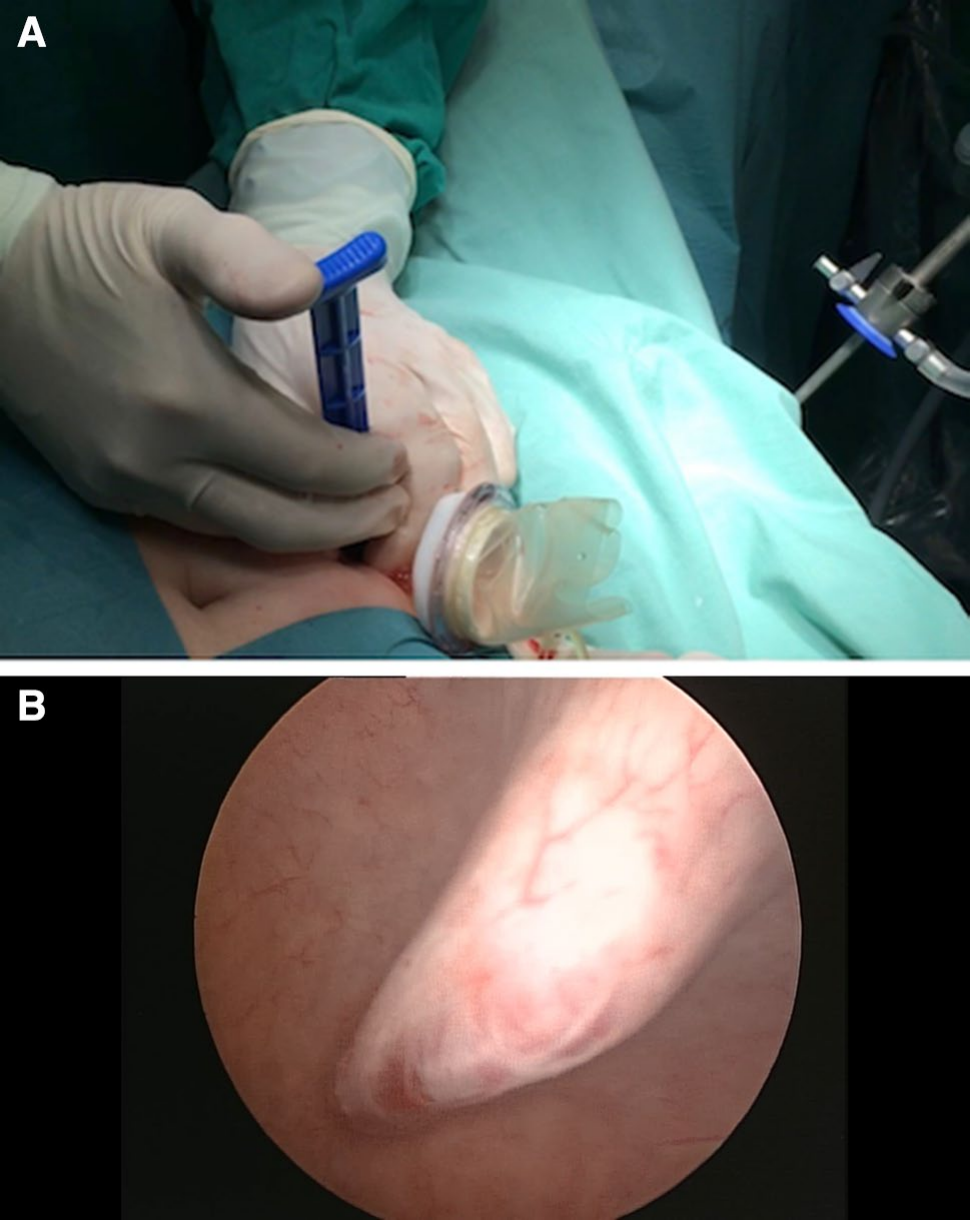
Percutaneous suprapubic establishment of the Tri-Port+ device. **a** Introduction of the Tri-Port+ via 1.5 cm skin incision. **b** Blunt insertion of the port through the bladder wall

The surgery began with standard cystoscopy, when we filled the bladder with 300–400 ml of saline, depending on the volume of the bladder. A 15–25 mm skin incision was made 2 cm above the pubic symphysis. In most cases, an additional incision of rectus fascia was added. The fabric single-port (Tri-Port+, Olympus, Germany) (Fig. 2a, b) was introduced through the skin incision directly to the bladder under the control of the cystoscope, with no additional bladder wall incision. Such control makes the port insertion safer, because the tip of the port is followed visually when it is inserted into the bladder (Fig. 2a, b). No specific precautions were needed. When the trocar entered the bladder, the internal ring of the Tri-Port+ was pushed down. The trocar was pulled out, and both rings of the system were matched together and fixed to the abdominal wall. The saline was evacuated, and pneumovesicum was established with carbon dioxide up to 14 mmHg. In no procedure was the vesicouretero reflux of the gas observed.

The intravesical part of the mesh with fragments that were dissected from the bladder bottom were excised and removed.

**Fig. 3:**
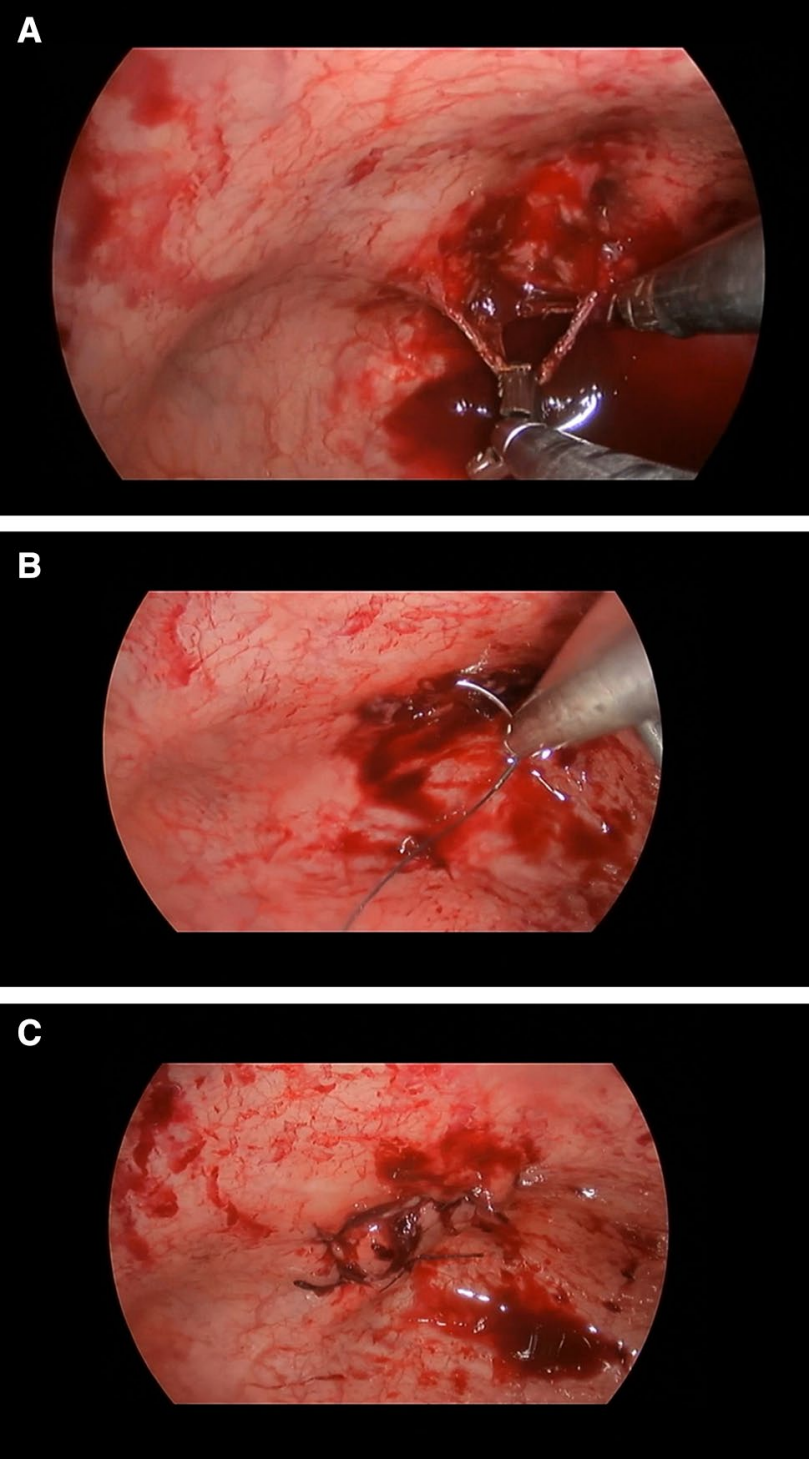
Operative steps. **a** Tape traction and dissection with standard laparoscopic instruments. **b** Bladder wall defect closure with running stitch. **c** View after suturing

In six patients, the bladder wall defects were closed with the running stitch V-Loc (Covidien, USA) (Fig. 3a–c). The rectus sheath and skin were sutured with two stitches. Foley’s catheters were introduced transurethrally and left for 5–9 days. Antibiotic prophylaxis (fluoroquinolones and cephalosporins) was administered before and after surgery. The period of therapy depended on the active or persistent urinary tract infection, based on the results of the microbiology examination.

In all operations, transurethral access was used for a grasper or suction tube. A visual analog scale was used to assess post-operative pain. As part of the follow-up interview, abdominal ultrasonography, general examination, and urine culture were performed in 6 weeks and 6 months after surgery. Ultrasonography was used to exclude complications such as pelvic hemorrhage, hydronephrosis, and stone formation. Moreover, the patients—except for those who refused the examination—were examined with cystoscopy.

A search was performed in a PubMed base using key words “mid-urethral sling”, “transvaginal mesh”, “mesh erosion”, “bladder erosion”, “single-port laparoscopy”, and “laparoendoscopic single-site surgery” for all available literature in English, from January 2005 to March 2018. Articles were selected to fit the scope of the topic, i.e., dealing with the management of tape/mesh erosion of the bladder or urethra. A total of 27 articles were identified, among which 6 papers presented the percutaneous transvesical access.

## Results

All procedures were successful, and no additional port was used. Conversion to the open method was not necessary in any of the cases. The average operation time was 54.5 min (range 23–135 min), and the average post-operative hospitalization was 30 h (range 8–96 h). Blood loss was minimal in all procedures. Patients received oral meals on the first post-operative day.

During the 6-month follow-up (range 3–10 months), there was no recurrence of erosion in the bladder, although in Patient 3 we observed an extrusion of the residual tape in the urethra. This was a consequence of an incomplete excision of the eroded calcified tape during the transurethral surgery performed before referral to our center. In this patient, the T-LESS access to the urethra was inefficient, because, after excision of the tape, we were not able to suture the opening of the urethral wall, and we only coagulated the bleeding vessels. We would like to underscore that this condition was asymptomatic and was found in the control cystoscopy.

None of the patients presented a positive urine culture in the follow-up. Moreover, symptoms like recurrent urinary incontinence or urge de novo were not observed.

The results of the literature review are presented in Table 2, and explained thoroughly in the "Discussion" section.

**Table 2:** Literature on suprapubic one-port mesh removal reported from January 2005 to March 2018 (*N* = 6)

Author (year of publication)	Number of pts	Initial surgery	Access/port placement	Mean operative time (min)	Post-operative stay (days)	Catheterization time (days)	Recurrence	Follow-up (months)
Al-Badr and Fouda (2005) [25]	1	TVT	Single-port	No data available	No data available	3	No	1.5
Ingber et al. (2009) [12]	2	No data available	Single-port (Tri-Port single-port access system)	113	0,8	7	No	7.0
Bekker et al. (2010) [26]	1	Prolift	Multiport	31	No data available	14	No	1.5
Yoshizawa et al. (2011) [15]	2	TOT	Multiport	146	No data available	7	No	18.0
Kim et al. (2012) [13]	3	TOT	Multiport	No data available	No data available	7	No	6.7
Roslan et al. (2013) [28]	9	TVT 2 Gynemesh 2 TOT 1 Prolift 1 Etc 3	Single-port (Tri-Port+)	59	2	5.9	1 (6 months after Prosima mesh removal)	18.8

## Discussion

Urinary incontinence is a very common problem, and affects about 50% of women at some point in their lives [16]. Of the patients undergoing polypropylene tape insertion for stress urinary incontinence (SUI), 9.8% experienced a complication relative to the procedure [16]. Complications like bladder or urethral erosions are the most challenging to manage. Bladder mesh erosions present as total or partial residence of the tape or mesh in the bladder wall, usually penetrating the bladder cavity and covered by calcifications [17].

Although most authors agree that removal of eroded materials is necessary, there is no consensus as to which method is preferable [9, 18, 19]. Authors presented isolated cases of bladder erosion in small numbers of patients [4–8, 20], Erosions are usually removed using the open technique. This approach reduces the symptoms significantly, but is invasive, and may cause the recurrence of urinary incontinence [2, 20].

The method of transvesical laparoscopic mesh excision from the bladder was presented by Sarlos [22], who treated 7 patients, and showed that the method is effective and safe. Rouprêt et al. reported on the removal of laparoscopic eroded materials from 9 females, and showed that this method is safe and feasible, but that in some cases leads to an increased risk of recurrent incontinence. They also concluded that removal of the entire implanted material is not necessary and may not affect the continence status of the patient [18].

To remove bladder erosions, various transurethral endoscopic techniques were applied, with the use of either laser- or electrocautery. These approaches are minimally invasive, with a short hospital stay, but they are associated with a significant rate of recurrence [23, 24].

Both open and laparoscopic approaches seem to be too invasive for the removal of relatively small fragments of implanted materials. The duration of hospitalization exceeds 6 days, and in some cases complications occur [18]. Al-Badr and Fouda excised successfully the part of the TVT tape penetrating the bladder with scissors that were introduced through a laparoscopic port directly into the bladder [25]. Bekker et al. followed the technique of Al-Badr, and also cut out the eroded mesh without suturing the bladder muscle layer defects [26]. To close the bladder wall defects with sutures, Yoshizawa et al. and Kim et al. used the transvesical access for multiport (3) laparoscopy and removed the penetrating materials [13, 15].

Ingber et al. in 2009 and Roslan et al. in 2011 were the first to perform the transvesical laparoendoscopic single-site surgery to remove bladder mesh erosions [12, 27]. The series of nine patients treated by this approach was presented in 2012 by Roslan et al. [28]. The authors achieved good results, with recurrence in only one patient. The mean operative time was 59 min and the hospital stay was 2.4 days [28].

These results encouraged us to introduce this innovative technique that seemed to be attractive because of its minimal invasiveness. As we are a tertiary referral center, we had the experience of several cases with bladder erosions that we operated on with open surgery. We noticed this type of access to be too excessive compared to the real severity of the disease. Thus we decided to change the approach for excision of bladder penetrating meshes, and finally to ensure that the new transvesical single-port procedure can be a valuable and reliable option to solve problems with bladder erosions.

We therefore applied a less invasive method that enables either the removal of eroded materials or the suturing bladder wall defects.

In our patient pool, the results were similar to those presented by other authors (Tables 1, 2), although in our group the hospitalization time was shorter by a factor of 2 than in previous reports. A hospital stay of 30 h and an operative time of 54.5 min confirm the minimal invasiveness and safety of the method.

In our group, cystoscopy was performed in 6 women. Pt 2 and Pt 6 refused the examination because of lack of symptoms.

The T-LESS procedure is characterized by excellent visualization of the operating field in the bladder, and the feasibility of intravesical suturing, which is not possible to perform via transurethral access. Additional advantages are the minimal post-operative pain and the possibility of using standard laparoscopic instruments that can be inserted transurethrally, which simplifies the procedure and shortens the time of surgery. We assume that the closure of the bladder wall defect may reduce the risk of fistula formation.

Another issue is the length of time for bladder catheterization. In our group, the duration of catheter maintenance depended on the type of removed material the width of the defect of the bladder wall.

Finally, we assume that leaving the urinary catheter for 5 days is sufficient for patients with small or stitched openings in the bladder wall. In addition, the distance from the patient’s place of residence was taken into account when determining the time of catheterization.

The limitations of this work are the small number of patients and the short follow-up time. We did not have a comparison group, because the described condition is rather uncommon, and we concentrated on the applicability and safety of the T-LESS technique.

We realize that this method is still innovative and its usefulness has not been evaluated fully. Nevertheless, the approach presents the potential to decrease the invasiveness and recurrence rate of the treatment of this deteriorating condition.

Thus, only multicenter cohort results may produce more reliable results; however, our experience suggests that the T-LESS procedure may be an attractive solution to the problems of open or laparoscopic procedures.

## Conclusions

Transvesical laparoendoscopic single-port removal of penetrating surgical materials from the bladder is a safe, effective, reproducible, and minimally invasive technique. This procedure offers a relatively short operative time, fast convalescence, and good cosmesis, and allows the use of either standard or sophisticated laparoscopic instruments. This method should be considered particularly when the local presentation of the foreign body is not suitable for endoscopic procedures or requires intravesical suturing. However, further studies, experience and development of new technologies are needed to facilitate the more widespread establishment of this method.

## References

[1] Ulmsten U, Petros P (1995). Intravaginal slingplasty (IVS): an ambulatory surgical procedure for the treatment of female urinary incontinence. Scand J Urol Nephrol.

[2] Cosson M, Caquant F, Collinet P et al. Prolift mesh (Gynecare) for pelvic organ prolapse surgical treatment using the TVM group: a retrospective study of 687 patients. Communication in the ICS Meeting Montreal, 31 Aug 2005

[3] Peyrat L, Boutin JM, Bruyere F (2001). Intestinal perforation as a complication of tension-free vaginal tape procedure for urinary incontinence. Eur Urol.

[4] Sergouniotis F, Jarlshammar B, Larsson PG (2015). Urethral complications after tension-free vaginal tape procedures: a surgical management case series. World J Nephrol.

[5] Parekh MH, Minassian VA, Poplawsky D (2006). Bilateral bladder erosion of a transobturator tape mesh. Obstet Gynecol.

[6] Forzini T, Viart L, Alezra E, Saint F (2015). Erosive complications of mid urethral slings (MUS): 10 years of surgical experience. Prog Urol.

[7] Wohlrab K, Erekson AE, Myers D (2009). Postoperative erosions of the Mersilene® suburethral sling mesh for antiincontinence surgery. Int Urogynecol J Pelvic Floor Dysfunct.

[8] Kasyan G, Abramyan K, Popov AA (2014). Mesh–related and intraoperative complications of pelvic organ prolapse repair. Cent Eur J Urol.

[9] Costantini E, Lazzeri M, Porena M (2007). Managing complications after midurethral sling for stress urinary incontinence. EAU-EBU Update Ser.

[10] Ahmed K, Ibrahim A, Wang TT (2012). Assessing the cost effectiveness of robotics in urological surgery—a systematic review. BJU Int.

[11] Rane A, Rao P, Rao P (2008). Single-port access nephrectomy and other laparoscopic urologic procedure using a novel laparoscopic port (R-port). Urology.

[12] Ingber M, Stein R, Rackley R (2009). Single-port transvesical excision of foreign body in the bladder. Urology.

[13] Kim JH, Doo SW, Yang WJ, Song YS (2012). Laparoscopic transvesical excision and reconstruction in the management of mid-urethral tape mesh erosion and stones around the bladder neck: initial experiences. BJU Int.

[14] Macedo FI, O’Connor J, Mittal VK, Hurley P (2013). Robotic removal of eroded vaginal mesh into the bladder. Int J Urol.

[15] Yoshizawa T, Yamaguchi K, Obinata D, Sato K, Mochida J, Takahashi S (2011). Laparoscopic transvesical removal of erosive mesh after transobturator tape procedure. Int J Urol.

[16] Ford AA, Rogerson L, Cody JD, Ogah J (2015). Mid-urethral sling operations for stress urinary incontinence in women. Cochrane Database Syst Rev.

[17] Haylen BT, Freeman RM, Swift SE (2011). An International Urogynecological Association (IUGA)/International Continence Society (ICS) joint terminology and classification of the complications related directly to the insertion of prostheses (meshes, implants, tapes) and grafts in female pelvic floor surgery. Int Urogynecol J.

[18] Rouprêt M, Misraï V, Vaessen C (2010). Laparoscopic surgical complete sling resection for tension-free vaginal tape-related complications refractory to first-line conservative management: a single centre experience. Eur Urol.

[19] Chan G, Mamut A, Martin P, Welk B (2016). Holmium:YAG laser ablation for the management of lower urinary tract foreign bodies following incontinence surgery: a case series and systematic review. J Endourol.

[20] Volkmer BG, Nesslauer T, Rinnab L (2003). Surgical intervention for complications of tension-free vaginal tape procedure. J Urol.

[21] Clemens JQ, DeLancey JO, Faerber GJ (2000). Urinary tract erosions after synthetic pubo-vaginal slings: diagnosis and management strategy. Urology.

[22] Sarlos D, Aigmueller T, Schaer G (2015). A technique of laparoscopic mesh excision from the bladder after sacrocolpopexy. Am J Obstet Gynecol.

[23] Ogle CA, Linder BJ, Elliott DS (2015). Holmium laser excision for urinary mesh erosion: a minimally invasive treatment with favorable long-term results. Int Urogynecol J.

[24] Barski D, Deng DY. Management of mesh complications after SUI and POP repair: review and analysis of the current literature. BioMed Res Int 2015;2015;831285 10.1155/2015/831285 PMC4418012 25973425

[25] Al-Badr A, Fouda K (2005). Suprapubic-assisted cystoscopic excision of intravesical tension-free vaginal tape. J Minim Invasive Gynecol.

[26] Bekker MD, Bevers RF, Elzevier HW (2010). Transurethral and suprapubic mesh resection after Prolift® bladder perforation: a case report. Int Urogynecol J.

[27] Roslan M, Markuszewski M, Gibas A (2011). Laparoendoscopic single-site transvesical removal of mid-urethral polypropylene sling eroded into the bladder. Videosurgery Other Miniinvasive Tech.

[28] Roslan M, Markuszewski MM (2013). Transvesical laparoendoscopic single site surgery to remove surgical materials penetrating the bladder: Initial clinical experience in 9 female patients. J Urol.

